# Children in All Policies (CAP) 2030 Citizen Science for Climate Change Resilience: a cross-sectional pilot study engaging adolescents to study climate hazards, biodiversity and nutrition in rural Nepal

**DOI:** 10.12688/wellcomeopenres.18591.1

**Published:** 2023-12-11

**Authors:** Katarina Hoernke, Aishworya Shrestha, Bhawak Pokhrel, Thomas Timberlake, Santosh Giri, Sujan Sapkota, Sarah Dalglish, Anthony Costello, Naomi Saville

**Affiliations:** 1 Children in All Policies-2030, University College London, London, WC1N 1EH, UK; 2 Institute for Global Health, University College London, London, WC1N 1EH, UK; 3 Kathmandu Living Labs, 1474 Lamtangin Marg, Chundevi, Kathmandu, Nepal; 4 School of Biological Sciences, University of Bristol, Bristol, BS8 1TQ, UK; 5 HERD International, Sainbu Awas Cr-10 Marga, Bhaisepati, Lalitpur, Nepal, Nepal

**Keywords:** "citizen science", Nepal, mobile app, climate hazard, biodiversity, nutrition, adolescents, "environmental stewardship"

## Abstract

**Background:**

Young people will suffer most from climate change yet are rarely engaged in dialogue about it. Citizen science offers a method for collecting policy-relevant data, whilst promoting awareness and capacity building. We tested the feasibility and acceptability of engaging Nepalese adolescents in climate change and health-related citizen science.

**Methods:**

We purposively selected 33 adolescents from two secondary schools in one remote and one relatively accessible district of Nepal. We contextualised existing apps and developed bespoke apps to survey climate hazards, waste and water management, local biodiversity, nutrition and sociodemographic information. We analysed and presented quantitative data using a descriptive analysis. We captured perceptions and learnings *via* focus group discussions and analysed qualitative data using thematic analysis. We shared findings with data collectors using tables, graphs, data dashboards and maps.

**Results:**

Adolescents collected 1667 biodiversity observations, identified 72 climate-change related hazards, and mapped 644 geolocations. They recorded 286 weights, 248 heights and 340 dietary recalls. Adolescents enjoyed learning how to collect the data and interpret the findings and gained an appreciation of local biodiversity which engendered ‘environmental stewardship’. Data highlighted the prevalence of failing crops and landslides, revealed both under- and over-nutrition and demonstrated that children consume more junk foods than adults. Adolescents learnt about the impacts of climate change and the importance of eating a diverse diet of locally grown foods. A lack of a pre-established sampling frame, multiple records of the same observation and spurious nutrition data entries by unsupervised adolescents limited data quality and utility. Lack of internet access severely impacted feasibility, especially of apps which provide online feedback.

**Conclusions:**

Citizen science was largely acceptable, educational and empowering for adolescents, although not always feasible without internet access. Future projects could improve data quality and integrate youth leadership training to enable climate-change advocacy with local leaders.

## Introduction

Climate change has been deemed the greatest threat to global health of this century ^1^, with children in low-and middle-income countries (LMICs) most at risk ^2^. Climate modelling predicts that, with current national climate policy pledges, children born in low-income countries (LICs) in 2020 face a fivefold greater risk of exposure to extreme weather events compared to those born in 1960 ^3^. In Nepal, it is estimated that children and youth make up more than 50% of the morbidity and mortality associated with climate change related disasters and face chronic malnutrition and stunting from associated agricultural losses ^4^.

Despite only historically contributing 0.01% to global cumulative CO _2_ emissions ^5^, Nepal’s average daily temperature is projected to rise more than the global average (ranging from 1.2°C to 4.2°C under the highest emission scenarios) by the 2080s ^6^. The country is particularly vulnerable to climate change, where the effects of intensified extreme weather events intersect with high rates of poverty, dependence on natural resources and subsistence agriculture. Unregulated road building and land development, combined with extreme weather events, contribute to landslides, flash floods and loss of biodiversity ^7, 8^. Rural areas are particularly at risk of food insecurity and loss of livelihood ^6^ given that the agricultural sector employs nearly two thirds of the population ^9^. As road access improves, the food system is shifting from subsistence agriculture towards food importation, exacerbating the dietary shift from healthier traditional foods to highly processed unhealthy foods ^10, 11^.

Limited national policies to tackle the impacts of climate change coupled with a lack of youth-targeted interventions in Nepal put children especially at risk ^12, 13^. The Child-centred Disaster Risk Reduction and Climate Change (CDCC) 2021 report highlights the lack of knowledge on the impacts of the climate crisis on youth in Nepal and the importance of their participation in climate policy planning for successful implementation ^4^. Citizen science (CS), which encompasses “a range of participatory models for involving non-professionals as collaborators in scientific research” ^14^, offers an opportunity to address these issues. The process of engaging citizens in scientific research can enable cost-effective and timely large-scale data collection whilst generating new and diverse perspectives, promoting personal and collective agency and building capacity of communities and policy makers to address a range of environmental and public health issues ^15, 16^. By valuing experiential and local knowledge, CS methods take a decolonising approach to climate change and health research ^17^, and can reach those normally excluded from science and policy discourse, including remote and marginalised communities ^18^. In the context of climate change, the approach can build “earth stewardship” by combining environmental education with natural history observation ^19^. CS has been applied to measuring air pollution ^20^, monitoring biodiversity ^21^ and vector-borne diseases ^22, 23^, as well as disaster risk reduction ^24, 25^. CS applications (apps) are being used to collect data, as well as relay health-related warnings and recommendations to users ^20^.

In Nepal, CS has been used to strengthen resilience to environmental disasters ^26^, including floods ^27^ and landslides ^28^. CS with Nepali youth has been used to monitor rainfall with school students ^29, 30^ and to collect geospatial data for open street maps (OSM) with university students ^31^. While the value of youth engagement in CS projects to promote awareness, agency and action on climate change has been demonstrated in high-income settings ^32– 37^, few studies have investigated CS with youth in LMICs ^38^.

The 2019 review by Chari e *t al.* demonstrated the need for more research on the advantages and disadvantages of CS projects to improve community preparedness and resilience to environmental disasters ^24^. To understand the role youth citizen scientists can play in climate change and health research, more studies are needed on the effectiveness and feasibility of youth as data collectors ^33^. Yet, to our knowledge, no studies have investigated the use of CS with youth to monitor climate change impacts on people and their environment in Nepal. To inform future work, we aimed to test the appropriateness and feasibility of CS related to climate change and health with adolescents in two contrasting districts in Nepal. We aimed to understand to what extent CS participation raised awareness about climate change and nutrition, resulted in usable data, and provided a foundation for youth climate advocacy and action.

This work is part of Children in All Policies 2030 (CAP-2030) ^39^ which implements the recommendations of the WHO-UNICEF-Lancet Commission report A Future for the World's Children? ^40^.

## Methods

### Research questions

Our research questions were:

1) To what extent is it feasible and acceptable for adolescents to use CS apps to gather climate-change and health related data in their local areas?
2) What are the challenges of using mobile technologies in the Nepalese context, comparing more accessible with very remote rural locations?
3) To what extent were Nepali adolescents aware about climate change issues before and after participating in CS activities?

### Study setting

**Figure 1.**
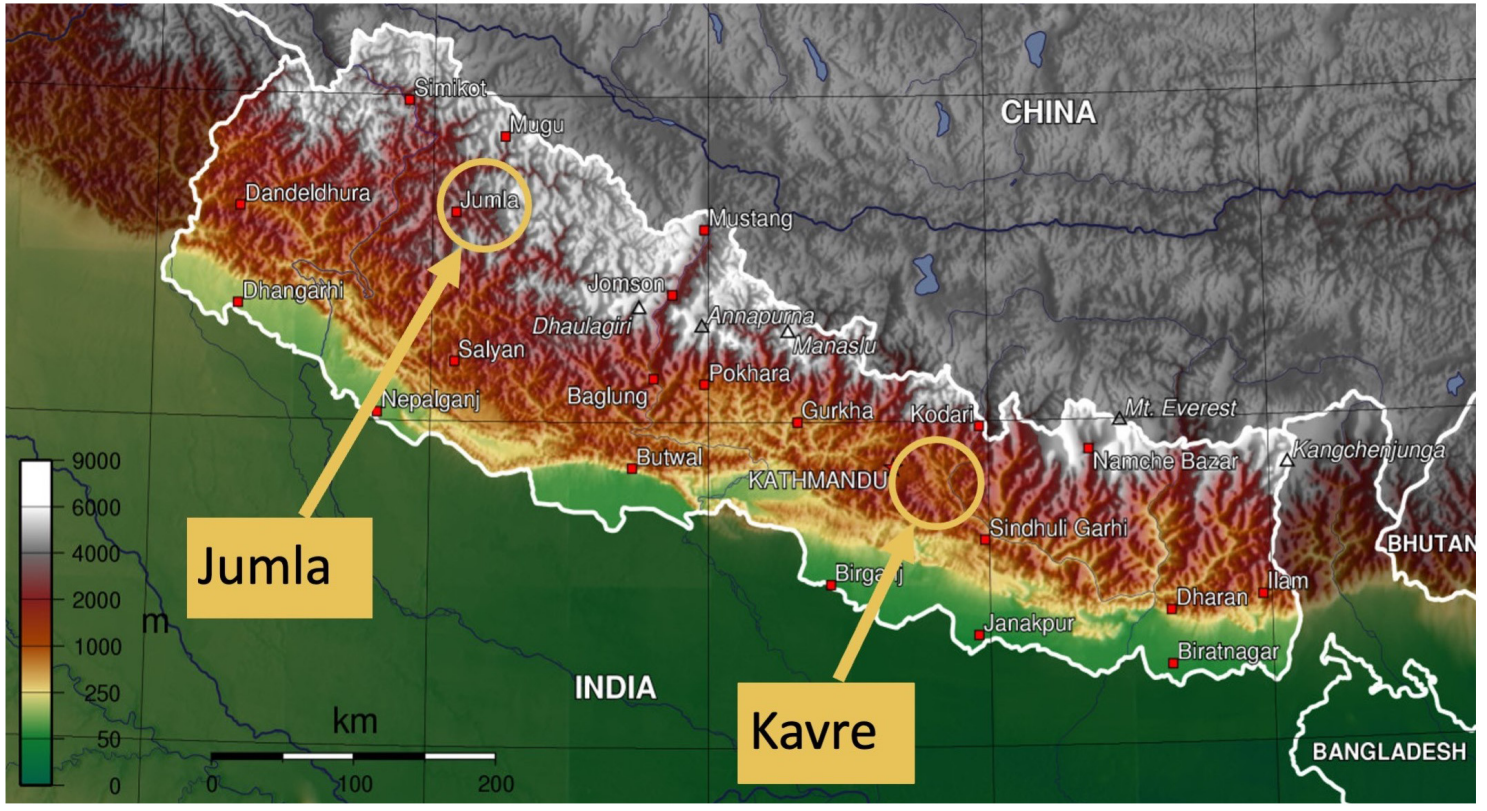
Kavre and Jumla districts (adapted from original image ^41^). *Note: As per the instructions of the copyright holder this file is licensed under the Creative Commons Attribution-Share Alike 3.0 Unported license. Permission is granted to copy, distribute and/or modify this document under the terms of the GNU Free Documentation License, Version 1.2*.

To conduct feasibility testing in areas with differing levels of mobile phone and internet connectivity in Nepal, we purposively selected two secondary schools in contrasting municipalities (Mandandeupur and Patarasi) in the relatively accessible mid-hill district of Kavre and the remote mountain district of Jumla respectively ( Figure 1). Jumla is one of the most inaccessible districts in Nepal (three days drive from Kathmandu) in Karnali province in western Nepal with altitudinal range from 915 to 4679 metres. Kavre is more accessible district (two to three hours’ drive from Kathmandu) in Bagmati province, with an altitudinal range of 280 to 3018 metres. Jumla and Kavre differ in terms of agroecological zone (mountains *versus* hills), mobile phone/internet access (very poor *versus* intermittent/adequate), and food insecurity and under-nutrition (severe *versus* moderate).

### Recruitment and consent

We introduced the project to community leaders, school principals and teachers in each municipality *via* stakeholders’ workshops. Students were eligible to participate if they had a keen interest in environmental issues and were studying in grades seven to 10 in our two selected schools. Participants included 13 students in Kavre, and 20 students in Jumla aged 12 to 16 years old, who volunteered to take part, after encouragement from their teachers. The number of participants were limited by the number of tablets (20) available to use the apps on.

Written informed consent for participation and publication of the participants details and their images was obtained from the parents/guardians of the participants after a meeting to explain the project.

In Kavre recruitment took place on 15 ^th^ to 16 ^th^ March before training from 4 ^th^ to 6 ^th^ April and data collection took place over several days between 5 ^th^ April and 19 ^th^ May 2022. In Jumla, recruitment, training and data collection took place between 13 ^th^ and 22 ^nd^ June 2022.

### App selection and creation

**Figure 2.**
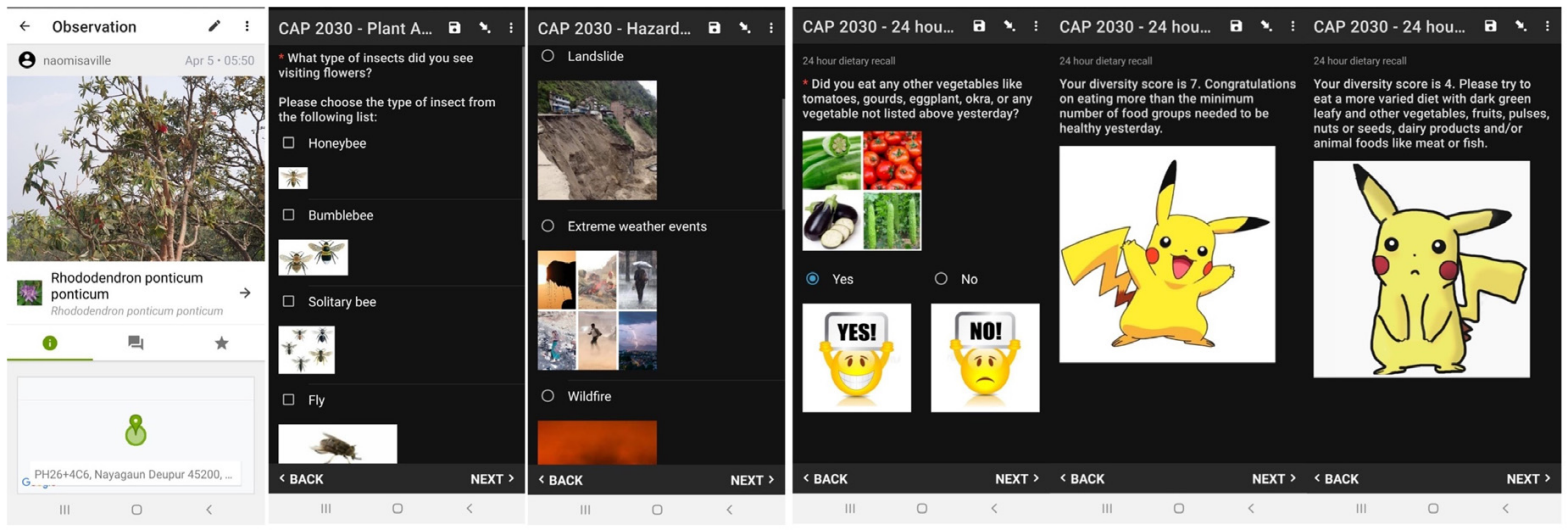
App interfaces (left to right): iNaturalist, Plant Atlas (pollinators), Hazard mapping, and Nutrition app dietary recall. *Note: Two feedback screens for adequate and inadequate dietary diversity scores are displayed*.

We identified three pre-existing open access apps which were user-friendly and appropriate to the study aims: iNaturalist ^42^, OSMTracker ^43^ and Organic Maps ^44^. We developed our own software to adapt and contextualise these apps for the study ^45, 46^. We also designed and tested four bespoke apps, using Open Data Kit (ODK), an open access mobile data collection platform ^47^. These apps record climate-induced hazards (Hazard mapping app), plants specific to the Jumla locality (Plant atlas app), 24-hour dietary recall and nutritional status (Nutrition app) and a sociodemographic survey of participants (Sociodemographic app). App design was targeted at adolescents by including photos and cartoon images. A summary of the apps is provided in Table 1 and details of the design features are given below. Images of the app screens are given in Figure 2.

**Table 1.** Summary of Citizen Science apps used in the study.

Name of app	Use prior to study	Use in this study	Type of data collected
Hazard mapping app	None	Created for this study with ODK to record climate change related hazards in the form of geolocations (crop pests, failed crops, inundating floods/flash floods or mudflows, landslides, wildfires, sinkholes and other unspecified hazards) and dates of the events.	Photographs, geolocations and geoshapes of hazards, dates of events.
Open Street Map (OSM) Tracker *#*	A GPS tracker for journeys that allows the user to mark geolocations and record tracks.	Translated into Nepali. Adapted to record geolocations of water sources, waste management and infrastructure.	Photographs and geolocations.
Organic maps # #	An open-source offline maps app based on OSM that allows users to add geolocations offline.	Used to increase general map literacy, locate and record geolocations of environmental points of interest.	Photographs and geolocations.
iNaturalist # ##	An online app used to collect and share biodiversity data (mainly plants and animals) by comparing a photograph of the observation with a digital database.	Translated into Nepali. Used to record and identify plants, insects, mammals, birds, amphibians, and reptiles.	Photographs, sounds, observations and geolocations of plants and animals.
Plant atlas app	Handbook used in the pre- existing ‘Micro-Poll’ research project based in Jumla. It contains local plants with English, Nepali and Scientific names and a QR code for each species. Users record plants by scanning the QR code.	Adapted for this study from Micro-Poll ‘CommCare’ questionnaires using ODK to identify plants, insect groups (potential pollinators, or pests) and plant diseases, by taking photographs and filling a form.	Photographs, observations and geolocations of plant species, insect flower and pests/diseases.
Nutrition app	None	Created for this study with ODK to record 24-hour dietary recall and/or anthropometry. Relays nutritional status and dietary results to users and gives tailored dietary advice.	Anthropometry (height, weight); recall of previous 24-hour consumption of 10 healthy food groups, 6 sentinel unhealthy food groups and tea drinking.
Socio-demographic app	None	Created for this study with ODK to record sociodemographic information and pre- existing knowledge of climate change and mobile devices amongst participants. Also used to train adolescents on using and collecting data through apps.	Sociodemographic factors, adolescent’s previous knowledge of climate change and environmental concerns, access to and use of mobile devices.

*Citations: #* Open Street Map. Learn OpenStreetMap Step by Step: OSMtracker *(2022); ##* Organic Maps OU. Organic Maps *(2022); ###* iNaturalist. Getting started with iNaturalist *(2022).*

***Design of the Hazard mapping app.*** We designed the Hazard mapping app using ODK for simple mapping of climate-change related hazards including inundation, landslides, extreme weather events, wildfires, flash floods or mudflows, sinkholes, cyclones, crop pests, failed crops and others (which the user may specify). Each hazard type can be selected by clicking a photographic icon, then the user may take up to three photographs of the event, record the geolocation, the date the event occurred (if known), the area covered by the disaster (using an online map) and remarks.

***Design of the Plant Atlas app.*** As part of the ‘Micro-Poll’ study on pollination and dietary adequacy in Nepal ^48^, TT, SS, NS and SG developed a printed photographic ‘Plant Atlas’ book containing photographs of many of the key pollinator forage species found in Jumla, together with their Nepali, English, and Scientific names and a QR code encoding these names and a unique identification code. They also developed electronic forms to collect data on plants and pollinators using the CommCare data collection platform. In this study, we adapted these tools to make a bespoke Plant Atlas app using ODK. This involves scanning plant QR codes to record plant observations found in the Plant Atlas and observing and recording diseases, plant pests, and insect visitors to flowers. Using Micro-Poll training materials, we generated pictorial answer options for insect visitors to flowers to differentiate between broad pollinator types (honeybees, bumblebees, solitary bees, butterflies, moths, wasps, flies, beetles). For recording plant pests, we used online photographic resources to make pictorial answer options for broad groupings of plant pests such as caterpillars, army fall worms (or similar), snails/slugs, beetles, and others. We made space for data collectors to capture photographs of the known and unknown plants, insect flower visitors, pests, and diseases.

***Design of the Nutrition app.*** The Nutrition app was designed for adolescents to collect data on the nutritional status of their local community (children and adults). In Kavre, the children measured one another and other members of their school community. In Jumla, with support from the research team, the children organized a nutritional assessment session at their school. The adolescents collected the data from respondents using the app and relayed results back to respondents on their dietary diversity, unhealthy eating score, thinness and/or height category, and associated health advice ( *e.g.* to eat more healthy food).

To calculate dietary diversity in the Nutrition app, we used the 10 food group minimum dietary diversity for women (MDD-W) Food and Agriculture Organisation (FAO) guidelines with a minimum cut-off of ≥5 groups for adults and children above five years old ^49^. As there is no established dietary diversity score for children above 5 years and the score for young children uses breast milk, we applied the MDD-W for all respondents above 5 years at enrolment. For young children we used the child dietary diversity score as defined in the updated 2021 UNICEF/WHO guidelines on infant and young child feeding ^50^), which we applied to all children six to 59 months old. This guideline contains breastmilk as a group (intended for children six to 23 months in age) so in this age-group, we used the cut-off of ≥5/8 food groups to indicate adequate dietary diversity. For children 24 to 59 months old, we used the cut-off of ≥4/7 groups, not including breastmilk. To calculate unhealthy food scores, unhealthy food groups were defined as per the WHO “sentinel unhealthy foods” which include the most common foods high in sugar, salt and/or unhealthy fats ^50^. These included: sugar sweetened beverages (packaged soda/juices), sugar sweetened tea/coffee, sweets/chocolate, deep-fried foods, instant/packet noodles, crisps/chips, and eating zero fruits or vegetables in the preceding 24-hours. These were added together in the app to make a seven-item score. Since the phytates (tannins) in tea prevent the absorption of important micronutrients, the app captures tea drinking behaviour in within an hour of mealtimes.

Using these scores, the Nutrition app generates basic individualised diet and exercise advice. A happy or unhappy cartoon image is displayed depending on whether the respondent’s dietary diversity is above or below the age-appropriate cut-off. If dietary diversity scores are below the cut-off, the app generates a message advising the respondent to eat more fruits, vegetables, animal foods, pulses, nuts and seeds. For unhealthy food scores, the score is reported back to respondents. If they score one or more, they are advised to avoid unhealthy foods and eat more fruits and vegetables.

For those providing height and weight data, the Nutrition app calculates BMI for each respondent as height and weight values are entered. It then uses the BMI, height, weight, and respondent age to generate categories and cross-refer to lookup tables. These are taken from simplified WHO 2006 growth charts for weight-for-height/length *z*-score and height-for-age *z*-score for 24 to 59 months old children and weight-for-age *z*-score for 0 to 59 month children. We used WHO 2007 growth charts for BMI-for-age *z*-score and height-for-age z-score for respondents five to 19 years old ^51^. The respective sex and age-specific look-up tables returned categorisation of weight-for-height/length (for children under 5 years old) and BMI-for-age z score (for those five to 19 years of age) to indicate relative thinness and whether the person was under- or over-weight. For adults (>19 years), the form generated a BMI category according to the WHO BMI standard (BMI <17.0: moderate and severe thinness, BMI <18.5: underweight, BMI 18.5–24.9: normal weight, BMI ≥25.0: overweight, BMI ≥30.0: obesity) ^52^. A summary was presented back to each respondent/carer. Those who were moderately or severely thin/underweight were advised to seek care at a nearby health facility and to eat more nutritious food. For children zero to 59 months old with Severe Acute Malnutrition (defined as weight-for-height/length *z*-score <-3) carers were advised that the child was dangerously thin and that they should be immediately taken to the nearest Outpatient Treatment Centre for treatment and supplementary feeding. For children and adolescents between two and 19 years old the height-for-age category and whether the child is stunted was generated. Carers of stunted children were advised to feed them nutritious foods more frequently and/or to encourage them to eat larger amounts and to take them for regular check-ups.

***Design of the Sociodemographic app.*** We designed the Sociodemographic app using ODK to capture the following domains of information about participants and to enable them to learn how to fill in a simple form with pre-coded single and multiple answer options with pictorial images to aid interpretation. Domains of information in the app include: sex, age, address, class studying in, caste, mother-tongue, religion, engagement in paid and unpaid work, household size, head of household, parental education, sources, sufficiency, quality and treatment of drinking water, asset ownership ( *e.g.* radio, TV, bicycle, motorcycle, vehicle, solar panel, computer, refrigerator, dish TV), access to phones and internet, use of internet, accessibility of smartphones, and knowledge of climate change.

### Training

We prepared training materials on climate change, how to read maps and how to use the apps and delivered two–three days training in each school. To enable use of the apps we taught the adolescents how to open the bespoke ODK ‘Citizen Science collect’ collection of apps, and select the form to be filled (Hazard mapping, Plant Atlas, Nutrition or Sociodemographic), or to open iNaturalist, OSMTracker or Organic Maps. For each app we demonstrated how to navigate the form, type text, select answers from pre-coded questions, record geolocations, take photographs, amend responses as needed, and save/submit the form after completion.

To use the Hazard mapping, OSMTracker and Organic maps we taught basic map navigation skills to enable the adolescents to record geolocations of points of interest. In the Hazard mapping app, areas affected by the hazard could also be recorded by drawing a polygon to represent the affected area on the map.

In the case of the Plant Atlas app in Jumla, we taught the adolescents how to look up the plants in the atlas, scan the associated QR code and how to identify and record categories of pollinator or plant pest and plant disease. This app contained detailed instructions for taking good photographs to enable plant identification / characterisation, so we demonstrated how to follow these instructions to take useful photographs of plants, insects, and disease.

For the Nutrition app, we trained the adolescents on how to take height to the nearest completed millimetre using a Leicester stadiometer, paying attention to methods of avoiding parallax and how to position the person correctly on the stadiometer with their head in the Frankfurt plane. To record weight using a Seca digital weighing scale we taught the adolescents how to position the scale on a flat surface and zero the scale before recording weight.

To reduce bias in data collection we encouraged adolescents to take records with care and to measure each individual only once, though this was more difficult when recording biodiversity and geohazards.

### Data collection

**Figure 3.**
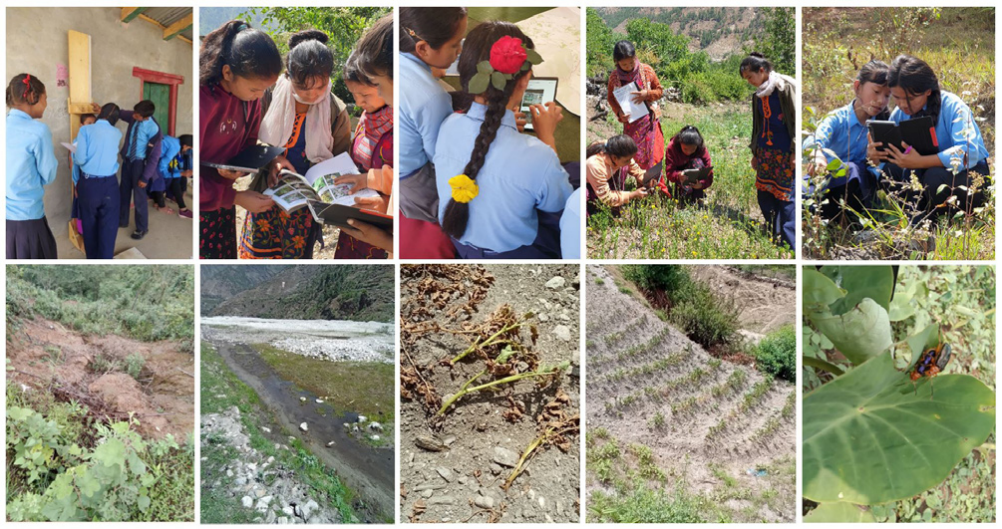
Participants taking anthropometric measurements, recording biodiversity using iNaturalist/Plant Atlas apps and recorded climate hazards. *Note: photo captions going left to right. Top row: a) taking height measurements; b) using the Plant Atlas and app; c) learning hazard mapping; d and e) recording biodiversity. Bottom row: f) landslide risk; g) flash flood location on previously cultivated land; h) insect pests on Taro; i) failed millet crop due to drought; j) failed potato crop. Written informed consent to publish these identifying images was obtained from the guardians/parents of the children shown*.

In each site the adolescents collected data for up to seven days, *via* guided data collection sessions at first, followed by independent data collection. In Kavre, after the initial training, the school science teacher managed the tablets and assisted the adolescents as needed. In Jumla, AS, BP and NS stayed in the community throughout training and data collection period, so were available to provide more hands-on support. Data collection covered: (i) details of landslides, floods, extreme weather events and crop pests/failure (geolocation and photos) *via* the Hazard mapping app; (ii) geolocation of waste management (rubbish dumps/bins), water sources and public amenities; *via* OSMtracker (iii) local plants, insects, birds and other organisms captured via photos/videos/audio (using iNaturalist) and (iv) crops/wild plants and associated insect flower visitors, pests and diseases using the ‘Plant Atlas’ and app; (v) dietary diversity and anthropometry of adolescents, adolescents and adults using the Nutrition app; (vi) sociodemographic details, climate change concerns and mobile phone access of participants using the Sociodemographic app (see Table 1). Participants mainly collected data in the vicinity of their schools but were able to take the tablets out of this area to their local villages to collect climate hazard and biodiversity data. Figure 3 shows photographs of students collecting data using different apps.

### Feedback and qualitative data collection

A formal longitudinal follow up was not feasible within this pilot study, however a feedback session was held. We downloaded maps of geolocations and sorted photographs to select observations of most interest to stimulate discussion. Using the plots, summary tables, maps and photographs we generated summary slide shows for each app in each school. We show-cased these to participants in data visualization workshops and discussed findings with them, linking observations with climate change wherever appropriate.

After discussing the findings, the AS, BP and NS held Focus Group Discussions (FGDs) with the adolescents to discuss their experiences, learnings, challenges, and recommendations with respect to each app and the overall process, using a topic guide which contained open questions to guide the discussion. None of the respondents knew the facilitators before the study but had become familiar with them over the days of interaction during the training and data collection. In Jumla we also conducted two FGDs to explore adolecents’ understanding of climate change and prior use of mobile devices before participation, so in Jumla most participated in a ‘repeat’ interaction, though with different questions. FGDs and the feedback sessions were held at the schools or, in the case of Jumla after the feedback session, at a locally hired hall. Most of the adolescents attended the focus groups, except one or two who needed to leave the session early. FGDs lasted between 23 and 75 minutes, apart from one very brief discussion during the wrap-up in Jumla, which took 10 minutes. FGDs were audio-recorded, and professionally transcribed verbatim and translated from Nepali to English. For transcription we sampled audio recordings to include material outlined in the topic guides and excluded discussions that fell outside these subjects.

At the end of the study, we presented certificates and (in Jumla) small prizes of stationery to the adolescents. We donated the 10 tablets to the school in Jumla.

### Data analysis

After data collection across each site, we synchronised (‘synched’) the devices. Data collected using pre-existing apps (from OSMTracker, Organic Maps and iNaturalist) were processed *via* the apps’ back-end data processing systems and also downloaded directly from devices onto a laptop. OSMTracker and iNaturalist summarised data in the form of a dashboard for iNaturalist and both apps generated maps showing the geolocations of different types of record.

BP downloaded ODK data as Microsoft Excel (version 16.71) spreadsheets or csv files from Kathmandu Living Labs’s server and iNaturalist and OSMTracker directly. NS and KH processed and analysed data using STATA version 17. For the Hazard mapping, Plant Atlas and Sociodemographic apps, we generated simple descriptive statistics. For the Nutrition app, we calculated dietary diversity and unhealthy eating scores and used the zanthro command to calculate anthropometric z-scores for height/length-for-age and BMIZ-for-age, for all below 19 years and z-scores of weight-for-age and weight-for-height/length for children under five years old, plotting results as bar charts.

Qualitative data were analysed using the Framework Method ^53^; a form of thematic analysis commonly used in multi-disciplinary health research that provides a systematic approach to analysing interview data. We used a largely deductive approach with mainly in vivo codes aimed at capturing the breadth of participants’ experiences but also some a priori codes derived from our literature review. KH and NS coded the transcripts using the software MAXQDA version 2022 (open source alternative: RQDA).

### Ethical approval

Ethical approval was gained from the Nepal Health Research Council on the 6 ^th^ December 2021 (611/2021P) and University College London’s Research Ethics Committee (No. 21579). Written informed consent for participation was obtained from the parents/guardians of the participants. Written informed consent was also obtained for the presentation and publication of identifiable images and photographs, as well as anonymised data such as participant characteristics and nutritional status.

## Results

### Participant characteristics from the Sociodemographic app

Table 2 provides sociodemographic information on the participants. In Jumla the 10 girls and 10 boys were mostly Hindu, Nepali speakers belonging to Chettri caste, aged 12 to 15 and studying in classes seven to 10. In Kavre the 12 girls and one boy were Buddhists from Tamang or Gurung castes, aged 13 to 16 and studying in classes eight and nine.

**Table 2.** Sociodemographic characteristics of adolescents and their households.

District	Jumla		Kavre	
**Total sample size**	n=20		n=13	
	*Mean*	*SD*	*Mean*	*SD*
**Total number of household members**	5.1	*1.4*	6.6	*2.4*
**Altitude of house**	2572	*62.7*	1518	*13.7*
	*Frequency*	*%*	*Frequency*	*%*
**Type of work**				
No work	10	50%	6	46%
Unpaid work	5	25%	3	23%
Paid work	4	20%	1	8%
Both paid and unpaid work	1	5%	3	23%
**Type of paid work**				
Agricultural	1	20%	2	50%
Teaching	3	60%	1	25%
Daily waged labour	1	20%	0	0%
Business	0	0%	1	25%
**Type of unpaid work**				
Fetching or carrying water	3	23%	3	21%
Cooking	4	31%	4	29%
Childcare	3	23%	0	0%
Livestock rearing	0	0%	3	21%
Farming and cultivation	0	0%	1	7%
Cleaning	3	23%	3	21%
**Highest paternal educational qualification**				
No formal education	7	35%	4	31%
Some / completed Primary school	3	15%	5	38%
Completed secondary school	4	20%	3	23%
Vocational qualification	6	30%	1	8%
**Highest maternal educational qualification**				
No formal education	10	50%	5	38%
Some / completed primary school	3	15%	5	38%
Completed secondary school	1	5%	2	15%
Vocational or higher qualification	6	30%	1	8%
**Available domestic facilities**				
Radio	11	52%	2	10%
Television	8	38%	7	35%
Bicycle	0	0%	3	15%
Motorcycle	0	0%	3	15%
Power (solar, inverter, generator)	1	5%	1	5%
Refrigerator	0	0%	1	5%
Computer	0	0%	1	5%
Television (cable, dish, IPTV)	0	0%	2	10%
No facilities	1	5%	0	0%

About half of adolescents in both sites engaged in some form of work (paid, unpaid or both). Paid work included teaching, agricultural, daily waged labour, and business. Unpaid work included chores such as cooking, cleaning, fetching water, childcare and livestock husbandry. Fathers were usually the head of the household. Most parents across both sites had either no formal education or did not complete primary school. In both sites, most reported having a clean and sufficient main source of drinking water *via* a private tap with piped water. Access to radio and/or television was common but most other assets surveyed were rarely owned. In Kavre several households owned bicycles, motorcycles, refrigerators, computers, and cable/dish/IPTV televisions but these were not found in Jumla.

Participants were largely able to collect accurate self-reported data, but some errors were detected. For example, despite most parents finding it difficult to sign the consent forms, participants in Jumla reported mothers as educated above secondary level.

Table 3 describes adolescents’ prior knowledge about climate change, as well as the environmental issues and effects of climate change most concerning to them, as reported in the socio-demographic questionnaire. In Jumla very little prior knowledge about climate change was reported compared to about half of adolescents in Kavre. Intensified weather events were amongst the most concerning effects of climate change in both sites, in addition to habitat destruction and the spread of vector-borne diseases in Kavre. In both sites, environmental issues of most concern to the adolescents were air and water pollution and poor waste management. Adolescents in Jumla reported having limited access to the internet and smartphones compared to those in Kavre. Digital literacy and access to digital technology was generally much higher in Kavre, especially following experiences with remote learning during the COVID-19 pandemic.

**Table 3.** Prior climate change awareness and access to internet and phones of adolescents.

District	Jumla		Kavre	
**Total number**	n=20		n=13	
	*Frequency*	*%*	*Frequency*	*%*
**Prior self-reported knowledge about climate change**				
Knows something about climate change	2	10%	6	46%
Knows very little about climate change	18	90%	7	54%
**Most concerning effects of climate change**				
Intensified weather events (heat, storms, floods, droughts, wildfires)	2	100%	5	42%
Habitat destruction (ecosystem collapse, biodiversity loss)	0	0%	5	42%
Spread of disease and pest propagation	0	0%	2	17%
Effects on humans (health, migration)	0	0%	0	0%
**Most concerning environmental issues**				
Air pollution	13	30%	9	25%
Water pollution	14	33%	4	11%
Climate change	2	5%	5	14%
Poor waste management	9	21%	7	19%
Overpopulation	1	2%	3	8%
Haphazard use of natural resources	2	5%	4	11%
Unmanaged settlements	2	5%	4	11%
**Available communication facilities**				
Landline	0	0%	0	0%
Push button mobile phone	16	64%	11	44%
Smartphone	6	24%	7	28%
Internet connection	1	4%	6	24%
No communication facilities	2	8%	1	4%
**Access to smartphone**				
Impossible to access from parents	4	67%	3	43%
Difficult to access from parents	1	17%	1	14%
Somewhat difficult to access from parents	0	0%	1	14%
Easy to access from parents	1	17%	1	14%
Very easy to access (owns own smartphone)	0	0%	1	14%
**Purpose of the smartphone**				
Internet	2	33%	5	33%
To pay bills	0	0%	1	7%
Banking services	0	0%	0	0%
Online maps and location services	0	0%	3	20%
Government services	0	0%	1	7%
Attend online classes	0	0%	3	20%
Cell phone (general use)	4	67%	2	13%

See references list for full sociodemographic dataset ^54^.

### Hazard mapping app findings

Adolescents used the Hazard mapping app to record 59 hazards in Jumla and 13 in Kavre. The frequency of records represents users’ interest rather than prevalence of the hazards, as the same location was recorded by multiple users. Crop-related hazards were more frequently identified in Jumla as there was an ongoing drought at the time of data collection. Table 4 provides a summary of hazards mapped. See references list for full dataset ^55^.

**Table 4.** A summary of climate-change related hazards identified through the hazard mapping app.

		Jumla		Kavre	
Total number of hazards observed		n=59		n=13	
		*Frequency*	*%*	*Frequency*	*%*
**Hazard type**					
Crop pests		4	7%	1	8%
Failed crops		24	41%	0	0%
Flash flood / mudflow		2	3%	0	0%
Landslide		28	47%	12	92%
Other		1	2%	0	0%
**Estimated event** ** year (self-reported)**					
2006		0	0%	2	17%
2015		1	2%	0	0%
2016		0	0%	1	8%
2018		1	2%	0	0%
2020		4	9%	1	8%
2021		6	14%	0	0%
2022		31	72%	8	67%

### OSMTracker and Organic maps findings

**Figure 4.**
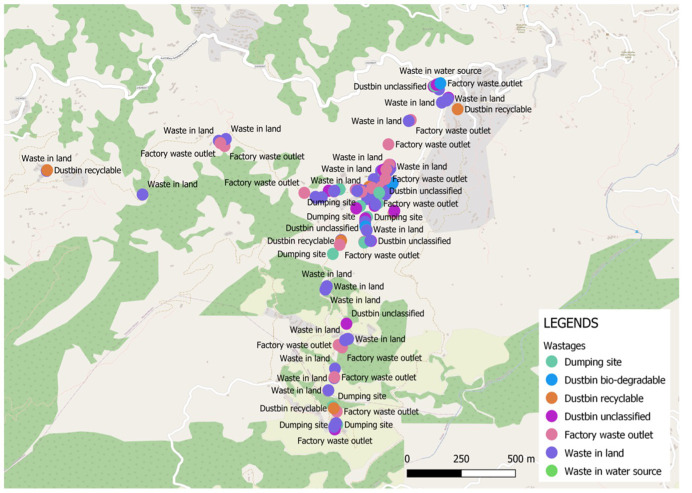
Waste disposal mapped in Kavre using OSMTracker app.

**Figure 5.**
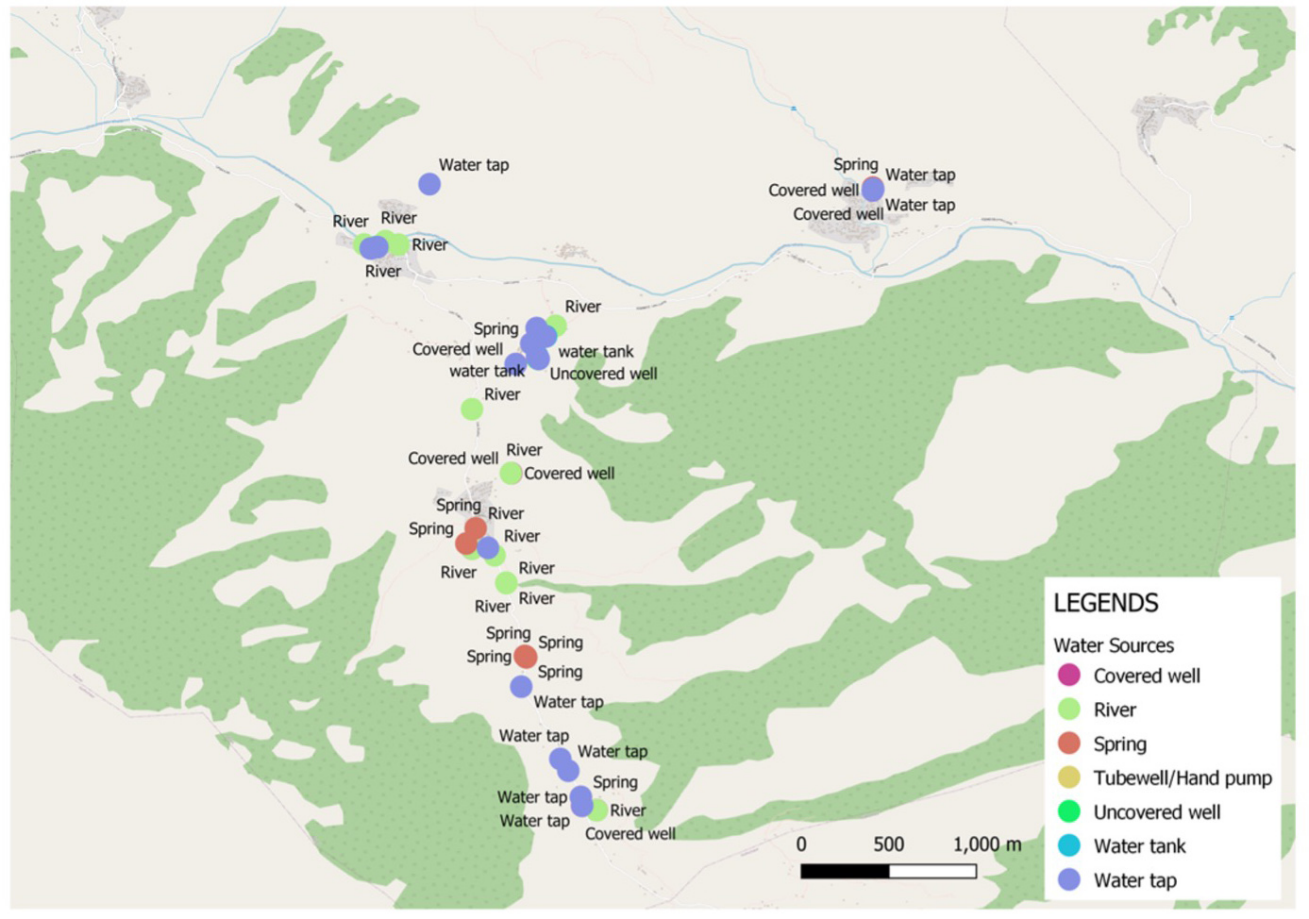
Water sources mapped in Jumla using OSMTracker app.

Adolescents recorded 223 and 421 geolocations using OSMTracker in Jumla and Kavre respectively, including waste disposal sites, infrastructure, and water sources. Frequency of geolocations recorded represents interest in recording the data rather than the prevalence of the geolocations themselves, since the same location may have been recorded on multiple tablets. Multiple locations of inappropriate waste disposal and dumping sites highlighted the need for better-managed waste disposal in the two municipalities ( Figure 4). The locations of water sources were well documented, though recording of the status of each source in terms of how well it was functioning or in terms of water quality could have added value. Participants used Organic Maps to develop navigation skills but not for saving data. Table 5 provides a summary of geolocations recorded using OSMTracker in Kavre and Jumla, and Figure 4 and Figure 5 the corresponding maps. See references list for full dataset See references list for full dataset ^56^.

**Table 5.** Summary of water, waste and infrastructure geolocations recorded using OSMTracker.

	Jumla		Kavre	
	n=223		n=421	
	*Frequency*	*%*	*Frequency*	*%*
**Category**				
Infrastructure	75	34%	118	28%
Waste disposal	100	45%	204	49%
Water Sources	48	22%	99	24%
**Infrastructure**				
Bridge	16	7%	5	1%
Buddhist temple	0	0%	1	0%
Bus stop	4	2%	40	10%
Police beat	1	0%	1	0%
Post box	0	0%	12	3%
Rest stop bench/tree	0	0%	14	4%
River	20	9%	19	5%
Road	22	10%	0	0%
Shop / Tea shop	0	0%	3	1%
Telephone	0	0%	1	0%
Toilets	4	2%	41	10%
**Waste disposal**				
Dumping site	17	8%	24	6%
Dustbin	22	10%	45	11%
Factory waste outlet	3	1%	43	10%
Waste on land	46	21%	87	21%
Waste in water source	12	5%	5	1%
**Water Sources**				
Covered well	10	5%	11	3%
Spring	15	7%	15	4%
Tube well/Hand pump	1	0%	0	0%
Water tank	2	1%	1	0%
Tap	26	12%	53	13%
Uncovered well	2	1%	0	0%

### iNaturalist findings

**Figure 6.**
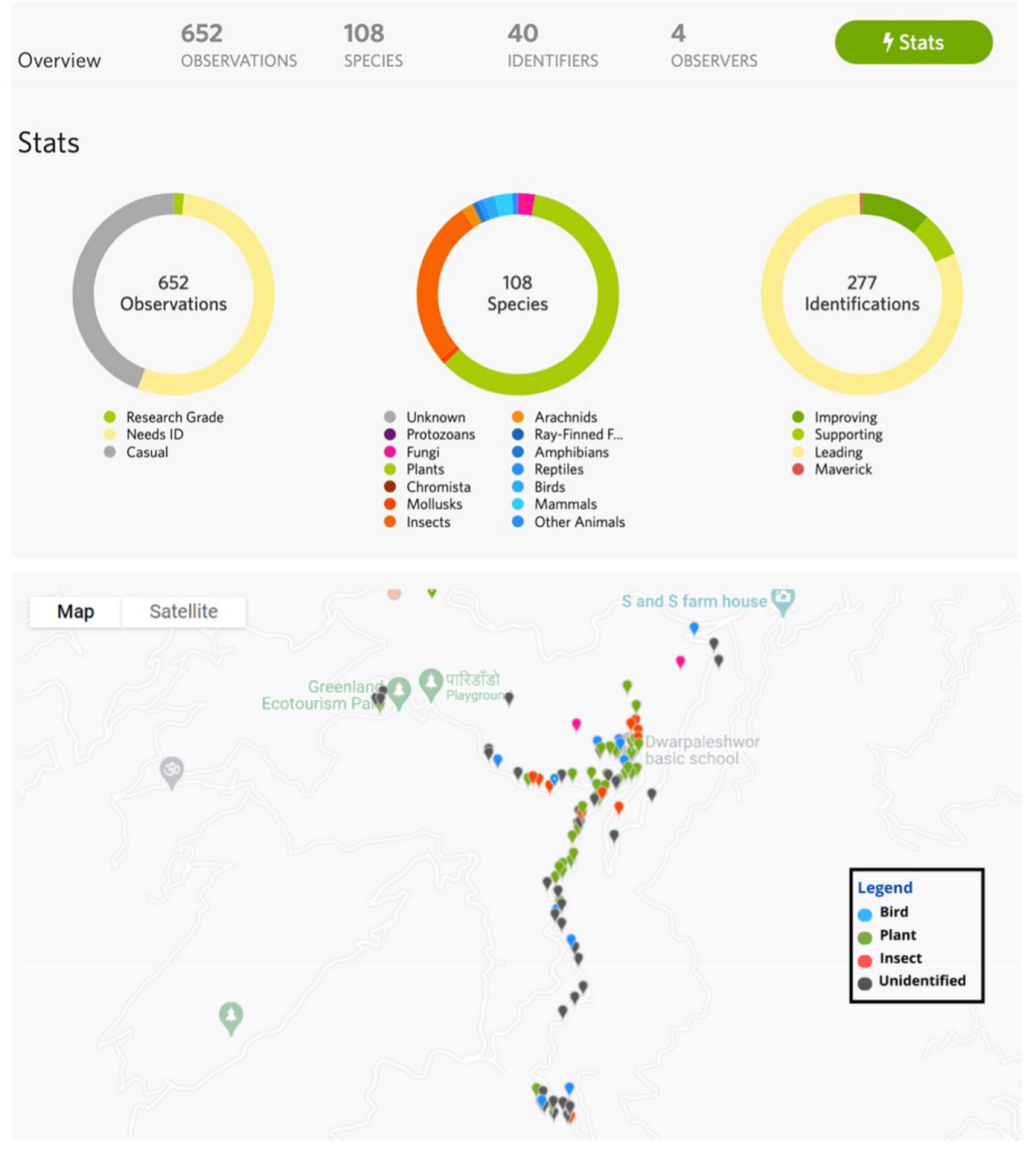
Data collected in iNaturalist in Kavre (dashboard and map as displayed in the app).

652 and 792 (total 1,444) observations were made in Kavre and Jumla respectively using iNaturalist. In Kavre, 277 observations were identified on the iNaturalist app ( Figure 6). A major challenge using this app was that it required internet connection to upload images and identify species/genera. This severely limited the usefulness of the app in Jumla, where tablets could not be synched at all on site. In Jumla, photos taken using the app were downloaded from devices and manually categorised by BP, AS and NS. A total of 298 animals (mainly birds and domestic animals) and 494 plants (crops, trees, wild plants) were recorded in Jumla ( Table 6). See references list for full dataset ^57– 59^.

**Table 6.** Summary of animals and plants recorded using iNaturalist in Jumla.

	Jumla	
	*Frequency*	*%*
**Total number of animals**	298	100%
**Total number of plants**	494	100%
**Animals**		
Algae	3	1%
Amphibians (frog spawn)	5	2%
Birds (chickens, wild birds)	33	11%
Insects		
Bees/wasps	56	19%
Butterflies/moths	45	15%
Others	114	39%
Mammals (domestic animals)	27	9%
Mollusca (snails, slugs)	10	3%
Reptiles (lizards)	2	1%
**Plants**		
Crops/cultivated plants	240	49%
Trees	78	16%
Wild plants	176	36%

### Plant Atlas app findings

Adolescents made 223 observations of crops/cultivated and wild plants using the Plant atlas in Jumla. Table 7 provides a summary of plants identified, as well as pollinators, pests and diseases observed on them. Table 8 lists wild plants and crops identified and frequency of observations. See references list for full dataset ^60^. Adolescents identified many of the same common plants as trained data collectors working in the nearby area on the Micro-Poll project, and they recorded similar relative abundances of different pollinator groups. Challenges, which pose risks to data accuracy and quality, included not all species being in the Plant Atlas and difficulty identifying and differentiating potential insect pollinators/pests. Recognising types of bees/wasps and flies and differentiating plant pests and diseases requires experience and skill. Adolescents were supported by experienced entomologists during training, but some participants nevertheless wished they had had more training.

**Table 7.** Summary of numbers of plants identified and pollinators, pests and diseases observed using the Plant Atlas app.

Plants and pollinators	*Frequency*	*%*	*n*
**Wild or crop plant**			233
Wild plant	102	44%	
Crop or cultivated plant	131	56%	
**Plant found in the Plant Atlas**	213	91%	233
**Flowers seen on plant**	110	48%	231
**Abundance of flowers**			
Rare (just a few individual flowers scattered throughout the area)	37	33%	
Scattered (appears in small numbers or isolated patches	18	16%	
Common (appears regularly throughout the area)	56	51%	
**Insects seen visiting flowers**	29	26%	111
**Type of insects seen on flowers**			29
Honeybee	7	24%	
Bumblebee	3	10%	
Solitary bee	4	14%	
Fly	12	41%	
Butterfly	5	17%	
Moth	0	0%	
Wasp	1	3%	
Beetle	4	14%	
Unknown	7	24%	
**Pests seen on plant**	44	19%	233
**Type of pests seen**			44
Caterpillar eating leaves	11	25%	
Aphid	6	14%	
Beetle	1	11%	
Grasshopper	4	9%	
Fall army worm (caterpillar eating roots)	4	9%	
Snail	1	2%	
Slug	1	2%	
Other	22	50%	
**Plants with disease**	36	16%	233
**Type of disease**			36
Fungal disease	4	11%	
Other plant disease	32	89%	

**Table 8.** List of wild plants and crops identified using the Plant Atlas book and frequency of observations.

Wild Plants				
English name	*Scientific name*	Nepali name	Total records	Edible plant (Yes/No)
Cheerful senecio	*Senecio chrysanthemoides*	Bijaure Phul / Malika Phul	9	N
Sikkim spurge	*Euphorbia sikkimensis*	Dhudya	7	N
Sagebrush	*Artemisia sieversiana*	Guiya paati	6	N
Dandelion	*Taraxacum sp.*	Gobe / Gobya	5	Y
Marsh marigold	*Caltha palustris*	Sim phool	4	N
Cannabis	*Cannabis sativa*	Bhango	4	Y
Ceylon forget-me-not	*Cynoglosum zeylanium*	Jhalo Kuro	4	N
White goosefoot	*Chenopodium album*	Bethe	3	Y
Wallich's thistle	*Cirsium wallichii*	Thakal	3	N
Wild Himalayan pear	*Pyrus pashia*	Mehel	3	Y
Mysore raspberry	*Rubus niveus*	Jogi Aniselu	3	Y
Indian willow	*Salix tetrasperma*	Baisa / Lahare Bais / Budhi Bais	3	N
Himalayan thyme	*Thymus linearis*	Ghoda Macho	3	Y
Stinging nettle	*Urtica dioica*	Sisnu	3	Y
Great mullein	*Verbascum thapsus*	Gunyu Puchhar	3	N
Anenome	*Anenome vitifolia*	Ban Kapas	2	N
Indian catmint	*Anisomeles indica*	Kale bhido	2	N
Himalayan fleece flower	*Bistorta affinis*	Makure Phul	2	N
Jimsonweed	*Datura stramonium*	Dhaturo	2	N
Balsam apple	*Momordica balsamina*	Ban karela	2	N
Silver-leaved cinquefoil	*Potentilla argyrophylla*	Pahelo Chaulya	2	N
Drumstick primrose	*Primula denticulata*	Neccabu	2	N
Cherry prinsepia	*Prinsepia utilis*	Dhatelo	2	Y
Two-flower raspberry	*Rubus biflorus*	Kalo Aniselu	2	Y
Long-leaved tansy	*Tanacetum dolichophyllum*	Bayojadi	2	N
Red bistorta	*Bistorta amplexicaulis*	Chyaau Phul	1	N
Himalayan strawberry	*Fragaria nubicola*	Bhuikaphal	1	Y
Hill geranium	*Geranium collinum*	Ratijara	1	N
Wallich geranium	*Geranium wallichianum*	Rakla mool	1	N
St John's wort	*Hypericum sp.*	Bheri Dale Phul	1	N
Figwort Picrorhiza	*Picrorhiza scrophulariiflora*	Kutki	1	N
Himalayan yellow raspberry	*Rubus ellipticus*	Aiselu	1	Y
**Crop or cultivated plants**				
English name	*Scientific name*	Nepali name	Total records	Food plant (Yes/No)
Potato	*Solanum tuberosum*	Aalu	12	Y
Maize	*Zea mays*	Makkai	7	Y
Pumpkin	*Cucurbita maxima*	Kaddu	6	Y
Sunflower	*Helianthus annuus*	Suryamukhi	6	Y
Apple	*Malus domestica*	Shyau	6	Y
Jumli bean	*Phaseolus vulgaris*	Jumli simi	6	Y
Mustard	*Brassica alba*	Tori	5	Y
Finger millet	*Eleusine coracana*	Kodo	5	Y
Apricot	*Prunus armeniaca*	Chule Aaru	5	Y
Cauliflower	*Brassica oleracea var. botrytis*	Kauli	4	Y
Chilli	*Capsicum sp.*	Khursani	4	Y
Soya bean	*Glycine max*	Bhatta (Kalo & Seto)	4	Y
Walnut	*Juglans regia*	Daatee Okhar / Kaate Okhar	4	Y
Dahlia	*Dahlia sp.*	Lahure	3	N
Common morning glory	*Ipomoea purpurea*	Lahare phul	3	N
Proso millet	*Panicum miliaceum*	Chino	3	Y
Peach	*Prunus persica*	Aaru	3	Y
Marigold	*Tagetes erecta*	Hajari	3	N
Wheat	*Triticum sp.*	Gahu	3	Y
Almonds	*Amygdalus communis*	Kaagati Badam / Madhesi Badam	2	Y
Coriander	*Coriandrum sativum*	Dhaniya	2	Y
Cucumber	*Cucumis sativus*	Kakhara	2	Y
Carrot	*Daucus carota*	Gajar	2	Y
Tomato	*Lycopersicon esculentum*	Golveda / Golbera	2	Y
Garden pea	*Pisum sativum*	Kerau mattar	2	Y
Pear	*Pyrus communis*	Naspati	2	Y
Onion	*Allium cepa*	Pyaz / Pyaaj	1	Y
Garlic	*Allium sativum*	Lasun	1	Y
Prince's feather	*Amaranthus hypochondriacus*	Marse	1	Y
Beet root	*Beta vulgaris*	Beet root	1	Y
Cabbage	*Brassica oleracea var. capitata*	Banda kovi	1	Y
Taro	*Colocasia esculenta*	Karkalo / Gaava / Pidhaalu	1	Y
Slipper gourd	*Cyclanthera pedata*	Chuche karela / Barela	1	Y
Tartary buckwheat	*Fagopyrum tataricum*	Tite phaaper	1	Y
Lambert's geranium	*Geranium lamberti*	Ratijara	1	N
Barley	*Hordeum vulgare*	Jahu	1	Y
Rice	*Oryza sativa*	Jumli Marsi Dhaan	1	Y
Himalayan peony	*Paeonia emodi*	Chandani	1	N
Perilla	*Perilla frutescens*	Tilkhudo / Tilkuro	1	Y
Daikon radish	*Raphanus sativus var.* * longipinnatus*	Mula	1	Y
Radish/ Sweet Turnip	*Raphanus sativus*	Choto / Koiro	1	Y
Foxtail millet	*Setaria italica*	Kaaguno	1	Y

**Figure 7.**
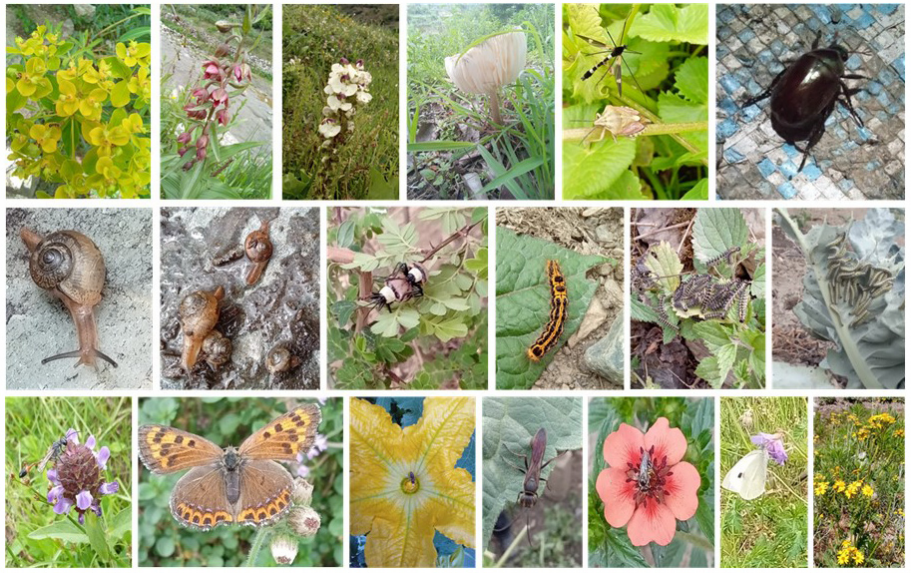
Photographs taken by adolescents of biodiversity (plants, fungi, and invertebrates including pests and pollinators).

A selection of photos of biodiversity taken by adolescents using iNaturalist and the Plant Atlas app is provided in Figure 7.

### Nutrition app findings

From the Nutrition app we present data of children/adolescents aged five–19 years and adults over age 19 years ( Table 9). More children were underweight in Jumla (17.3%) compared with 9.3% in Kavre, whereas stunting was more prevalent in Kavre (43.6%) compared with Jumla (25.5%). Only 2.3% were overweight in Jumla compared with 11.2% in Kavre.

**Table 9.** Anthropometry and dietary information from children 5-19 years and adults >19 years old.

	5-19 years old						>19 years old					
	Jumla			Kavre			Jumla			Kavre		
*Categorical Anthropometric outcomes*												
Stunting	n=137	Frequency	%	n=55	Frequency	%	n=14	Frequency	%	n=31	Frequency	%
Not stunted (5-19y) / ≥145cm (adults)		102	75%		31	56%		12	86%		28	90%
Stunted (5-19 y) / <145cm (adults)		35	26%		24	44%		2	14%		3	10%
BMI-for-age Z score / BMI category	n=133	Frequency	%	n=54	Frequency	%	n=14	Frequency	%	n=30	Frequency	%
Grade 2 thinness † / moderate and severe thinness *		2	2%		1	2%		0	0%		5	17%
Grade 1 thinness † / underweight *		21	16%		4	7%		0	0%		0	0%
Normal weight		107	81%		43	80%		12	86%		18	60%
Overweight		3	2%		5	9%		2	14%		5	17%
Obese		0	0%		1	2%		0	0%		2	7%
*Continuous Anthropometric outcomes*												
	n	Mean	*(SD)*	n	Mean	*(SD)*	n	Mean	*(SD)*	n	Mean	*(SD)*
Height-for-age z score	137	-1.3	*(1.0)*	55	-1.9	*(1.2)*						
BMI-for-age z score	135	-0.4	*(0.9)*	56	0.1	*(1.0)*						
Weight (kg)							22	52	*(7.1)*	33	56.6	*(-9.4)*
Height (cm)							14	149	*(5.6)*	31	160	*(14.6)*
Body Mass Index (BMI)							14	23.1	*(2.3)*	30	22.7	*(-4.6)*
*Dietary recall in last 24 hrs*												
	n=183	Mean	*(SD)*	n=67	Mean	*(SD)*	n=24	Mean	*(SD)*	n=42	Mean	*(SD)*
Age in years		13.5	*(2.5)*		14.5	*(2.7)*		36.8	*(12.3)*		31.5	*(12.1)*
Dietary diversity score #		4.7	*(2.1)*		5.5	*(1.8)*		5.1	*(2.0)*		5.7	*(1.7)*
Unhealthy eating score out of 7		1.8	*(1.4)*		3.7	*(1.5)*		1.2	*(1.2)*		2.4	*(1.4)*
Sex of respondent	n=183	Freq	%	n=67	Freq	%	n=24	Freq	%	n=42	Freq	%
Male		83	45%		19	28%		10	42%		18	43%
Female		100	55%		48	72%		14	58%		24	57%
Eating behaviour	n=183	Frequency	%	n=67	Frequency	%	n=24	Frequency	%	n=42	Frequency	%
Consumed sweet beverage		71	39%		41	61%		8	33%		27	64%
Consumed sentinel unhealthy foods (high sugar/salt/fat)		121	66%		65	97%		13	54%		33	79%
Consumed zero vegetables or fruit		22	12%		5	8%		1	4%		1	2%
24hr minimum dietary diversity (MDD) achieved		83	45%		44	66%		14	58%		31	74%
Tea drinking behaviour	n= 183	Frequency	%	n=67	Frequency	%	n=24	Frequency	%	n=42	Frequency	%
Does not drink tea/coffee		72	39%		32	48%		16	67%		21	50%
Drinks tea/coffee ≥1 hour either side of a meal		65	36%		16	24%		4	17%		13	31%
Drinks tea/coffee within 1 hour of a meal		46	25%		19	28%		4	17%		8	19%

Note: ^*^for adults >19 years old (as per WHO definitions of BMI categories in adult populations: BMI <17.0: moderate and severe thinness, BMI <18.5: underweight, BMI 18.5–24.9: normal weight, BMI ≥25.0: overweight, BMI ≥30.0: obesity ^52^) ^†^for children 5-19 years old (as per WHO definitions of BMI z-score categories in <5 years populations: grade 2 thinness: <-3 SD, grade 1 thinness: <-2 SD, overweight: >+1 SD, obesity: >+2 SD ^51^ ^#^ *MDD defined as ≥5/10 food groups for children ≥5 years & adults >19 years old*

**Figure 8.**
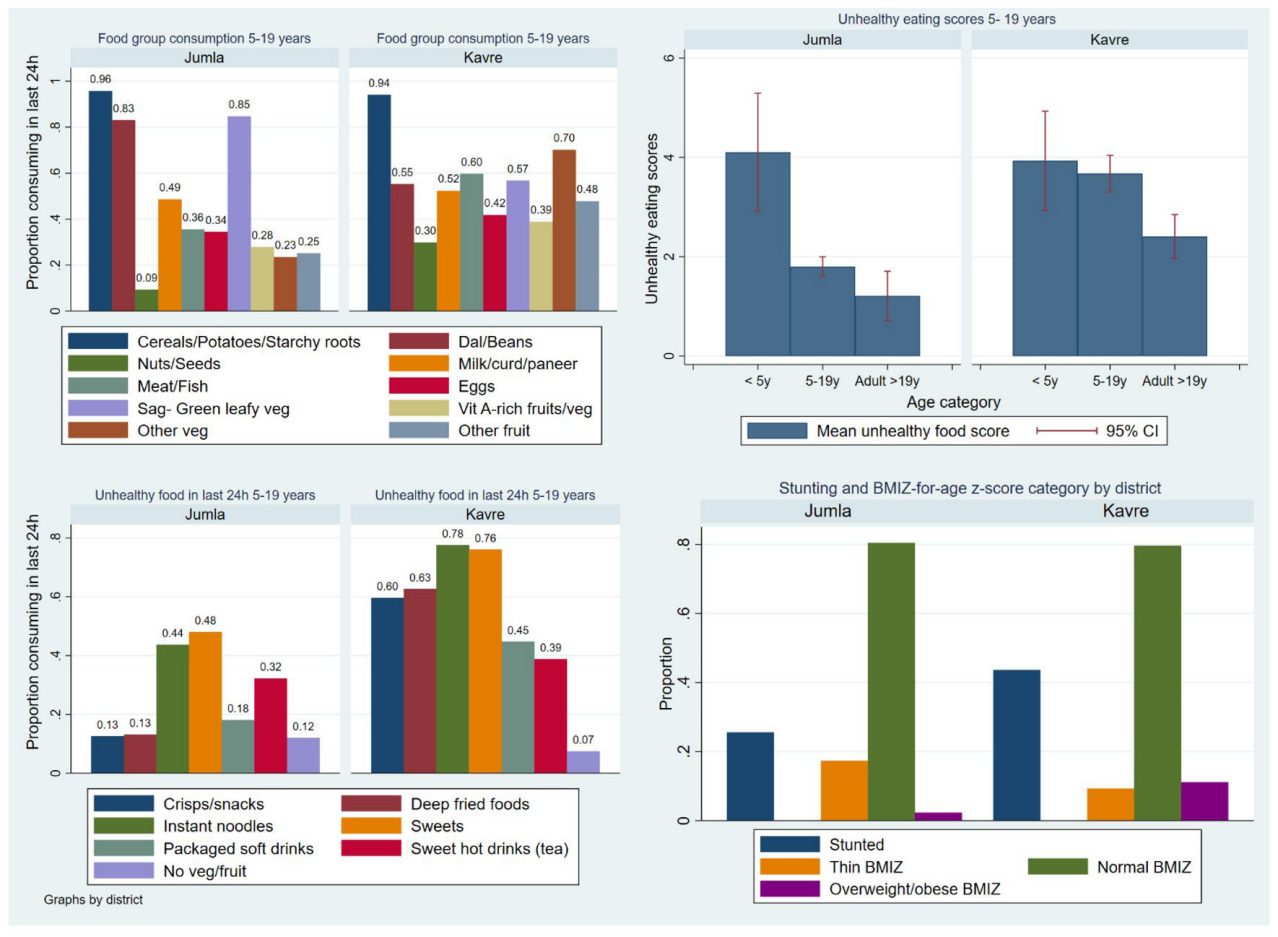
Healthy and unhealthy food consumption, BMI-for-age-z scores and stunting for children aged 5–19 years old. *Note: Clockwise from top left comparing the districts of Jumla and Kavre: **a**) consumption of 10 food groups; **b**) proportions stunted and in BMI-for-age z score categories; **c**) consumption of unhealthy sentinel foods by 5–19 year-old children; **d**) mean unhealthy eating scores out of 7 at different age groups with error bars representing the confidence interval*.

Unhealthy food consumption was higher in five- to 19-year-olds compared to adults >19 years ( Figure 8). Youth aged five–19 years in Kavre had higher adequate dietary diversity (66% vs 45%) than Jumla, but also higher unhealthy eating scores (3.7 [1.5 SD] *versus* 1.8 [1.4 SD]) ( Table 9). For five- to 19-year-olds in Jumla the main food groups consumed were cereals, pulses, and green leafy vegetables (GLV), followed by dairy. Fewer than 40% accessed meat/fish, eggs or other types of fruit or vegetable than GLV. In Kavre, around 40% or more consumed most food groups. In both districts, especially Jumla, nuts and seeds were consumed rarely ( Figure 8). Results from both locations (especially Jumla) reflect a monotonous diet with limited intake of micronutrients. Results from under five years old were not included due to the small sample sizes and inconsistent data collected.

As we did not have a stadiometer for measuring length in a supine position, only heights of children over 87cm could be taken, not lengths. Measurements could only be taken at the schools as equipment was not available for students to take home or into the community. Some anthropometric measurements were implausible and had to be discarded ( Table 9). Height-for-age calculations were more error prone, especially if children reported their age in running rather than completed years. This was more of a problem in Kavre than Jumla since data collection in Jumla was more closely supervised. With regards to the dietary diversity recording, adolescents in Kavre recorded unusually large numbers of food groups, which was difficult for the team to validate. For example, unusually, more than half the children under five years old in Kavre were reported to eat all the food groups, which may need validating. See references list for full dataset ^61^.

### General implementation experience

We found that having a pre-existing partnership and a good rapport with the schools improved acceptability amongst principals, teachers, and parents. Having buy-in with the school principal and teachers was important for the data collection process. We had to work around school schedules ( *e.g.* upcoming exams) and found that advanced planning and good communication significantly helped in one study setting compared to the other.

### Qualitative findings

Three themes emerged from the qualitative data: existing knowledge and experiences of climate change, lessons learnt and opportunities, and challenges with the apps and technology.

***Theme 1: Knowledge and experiences of climate change.*** Most adolescents exhibited some prior knowledge of climate change either through lived experience or (in Kavre) as part of their school curriculum. Understanding of the science behind climate change was largely lacking, especially in Jumla. There was some confusion between the causes and effects of climate change. Potential causes of climate change and environmental degradation mentioned included anthropogenic factors such as inappropriate waste disposal, deforestation, industrialisation, and use of poor-condition motorised vehicles.

*“After floods and landslides, our crops are also damaged. When there is a flood, our houses are washed away, and our agricultural lands are washed away.” (Boy, Jumla)*

Participants could describe their experience of climate change in the form of extreme weather events and changing seasonal patterns. Adolescents in Kavre were especially concerned about landslides, whereas in Jumla droughts, reduced rain or snowfall and failed crops were more common. Some discussed links between droughts, failed crops and diets.

*"The main thing now is we don't see plants and insects like before anymore [...] the flowers that were found earlier, and the grains that were found earlier are not found now. All of them have disappeared." (Adolescent, Jumla)*
*"The mustard seeds planted on our land have dried up […] by not getting rainfall in time. We don't get to eat green leafy vegetables.” (Boy, Jumla)*

They discussed a gradual dietary shift away from consumption of home-grown fresh vegetables and grains to purchased polished white rice (in Jumla) and maize (in Kavre). Some also described lower milk production due to reduced fodder for livestock.

***Theme 2: Lessons learnt and opportunities.*** Adolescents enjoyed using the apps and appreciated the opportunity to learn about their local environments with respect to biodiversity, crop failure and climate hazards like floods and landslides.

*“It was very fun; this day will never come again… Before this we had only seen* [the biodiversity] *… but this time we were able to capture photos as well. It was fun to walk around and take pictures of flowers and butterflies” (Adolescent, Jumla).*

After the data collection period, they expressed greater awareness about the impacts of these on their own communities, including links to agricultural food production and diets. They especially enjoyed photographing local insects and plants and learning about pollinators and pests. Many found the support and training from the research team stimulated their interest and understanding of how to use the apps.

*"...at first we weren't very much interested in it... but because of you all, we learned something and understood something, and we knew what is possible to do from this... It was a pleasure to learn." (Adolescent, Jumla)*

Adolescents appreciated the opportunity to learn about their health (BMI and height) and their diets through use of the Nutrition app. They learnt about anthropometry and dietary diversity, including the difference between healthy and unhealthy food groups, and how more "junk food" was consumed by young people than adults. In Jumla in particular, they recognised the health benefits of eating organically grown local foods. Some discussed learning about health conditions linked to nutrition ( *e.g.* diabetes, cancer, obesity).

*“We also learned that if we eat junk food, our body will not get the nutrients it needs.” (Boy, Jumla)*
*“We learned about health and that junk food should be reduced. If the consumption of junk food is high, we will be malnourished.” (Boy, Jumla)*

Through use of the Hazard mapping app, they became more aware of impacts of climate change *via* extreme weather events causing landslides and floods. In Jumla especially, students captured the consequences an ongoing drought upon crops using the hazard and Plant Atlas apps. This enabled them to start linking environmental changes to agricultural production and their dietary patterns.

*“From the technology of the tablet, we came to know whether the plant has died [from drought]. If the plants are drying from drought, the humans will suffer. The human body cannot get the required nutrition then.” (Adolescent, Jumla)*

Participants learned about how apps can be used to monitor environmental changes, especially after the data visualisation learning sessions. While there was no scope to capture changes in biodiversity over the short data collection period, adolescents said that using the apps stimulated their interest in learning about the nature around them. Many students knew little about pollination before the study and, in Jumla, enjoyed learning to differentiate pollinator types and crop plant pests.

*“I could see which insects are destroying the crops and farms (Adolescent, Jumla)”*

Some participants particularly enjoyed accessing information from the apps, such as identifying their nutritional status and getting basic diet and lifestyle advice free of charge. They were happy to support good health in their communities by offering this health advice to fellow schoolmates and villagers.

*“A person can't know exactly whether they are healthy or not, but with the help of a tablet we can know the right answer.” (Boy, Jumla)*
*“In terms of nutrition, how many people are healthy, how many people have less height ... or less or equal height according to age. I learned that as well.” (Adolescent, Jumla)*
*“We learned about what foods have effects on our body, and what foods to cut, and we learned that from the tab.” (Adolescent, Jumla)*

Most students saw value in collecting data on biodiversity and hazards to be able to address environmental issues. Students began demonstrating ‘environmental stewardship’ and an appreciation for maintaining their local environments.

*“[We can control climate change] by planting trees. We should reduce the use of vehicles. We should reduce the smoke emitted from vehicles. Old bikes and vehicles should be removed, and only new ones should be used. We should control garbage.” (Adolescent, Jumla)*

*"We learned ways to reduce garbage. Where is the garbage, where is pollution, and where is the garbage piled up, we learned how can it be reduced. We learned about the condition of the forest and about the insects that we will use. We learned to control pests that destroy crops and farms.*
*Interviewer: Why did you use the tab? For climate change? Why now?*
*Participant: … in order to make a change in our life. We shouldn’t kill plants and throw litter haphazardly... We should conserve everything..." (Boy, Jumla)*

One student even suggested bringing the results to their local municipality to show to government and policymakers.

*“After [taking photos] of hazards, we can visit our municipality and advise them regarding gaps that need to be addressed. (Boy, Jumla)”*

Being able to read and track locations on maps was empowering and exciting for students. Using OSMTracker, adolescents were able to understand their local environment better and were excited about the potential utility of maps to share their locations in relation to specific geolocations. Some also saw future opportunities for using maps in new places, enabling them to explore without fear of getting lost.

*“I found the organic map helpful because if we get lost somewhere in the jungle the organic map will show is the exact way” (Adolescent, Jumla)*

Other ‘favourite’ apps mentioned by the students were iNaturalist, Hazard mapping and the Plant atlas apps.

***Theme 3: Challenges with apps and technology.*** Some participants found it difficult to photograph insects or birds as they flew away or were frightened-off when the adolescents were trying to photograph them. In Kavre, some were demotivated by bad weather, stinging nettles, and being bitten by insects. One participant worried that other app users would be affected if she recorded incorrect data into OSMTracker app. While some participants wanted more time for data collection (especially in Jumla), others found it tiring to walk around in the hot sun collecting data for too long. Some participants were worried about crossing into unknown or private land.

*“When I went to the other farms, I was afraid that they would scold me. I crossed the barbed wire […] because I saw a new insect.” (Adolescent, Jumla)*

Some adolescents felt shy or awkward approaching adults to ask questions in the nutritional survey, so found if easier to use apps they could fill in without asking questions to others. They reported that certain members of the older generation were critical of the use of tablets and apps and did not see a value in collecting data.

*“…were collecting the data and suddenly his wife came and started asking us ‘what will you do just by filling up them it would be better if you would give us something to eat.’ ‘They don't do anything, they just collect the data, […] they don't offer any benefits.’" (Adolescent, Jumla)*
*"We don’t have enough time to ask others, they even don’t pay attention [...] Everyone is busy. And it felt awkward." (Girl, Kavre)*

In Jumla, we were able to interview students before training them on the apps, but in Kavre there was insufficient time for this. Adolescents in Jumla were familiar with mobile phones, mainly using them to play games and videos, call relatives, and sometimes for schoolwork to look up information. However, most used their parents' phones and did not own a phone of their own. A key barrier to mobile phone use in Jumla was lack of reliable internet connection. The almost complete lack of internet connection in the Jumla field site made using certain features of apps, and managing data, very challenging for participants. Users were able to synch data in the community in Jumla. Data had to be transferred to a computer *via* a wired connection for the ODK and other apps. Adolescents were unable to synch their data to the i-Naturalist community, so could not access species identification.

## Discussion

To our knowledge, this is the first study to test the acceptability and feasibility of engaging youth in CS methods to study the linkages between climate change, the environment, and nutrition in Nepal. We found that the approach was largely acceptable although not always feasible in rural and remote regions, primarily due to problems of internet connectivity. CS methods can be applied to monitor, and stimulate interest in, biodiversity, and to gather information on environmental and nutritional issues related to climate change. Adolescents enjoyed collecting data and the process raised their awareness about the impacts of climate change in their local environment, including landslides, floods, droughts, and failed crops. They identified over and under-nutrition, and poor diets in terms of low dietary diversity and high unhealthy eating scores. They learned how reduced production of home-grown micronutrient-rich food may drive increased consumption of unhealthy processed foods. The process of monitoring climate-change induced hazards and tracking biodiversity could be used to build ‘environmental stewardship’ to advocate for action with local leaders through participatory action and learning groups.

Limitations of this pilot study include small sample sizes, limited time for data collection, no field testing prior to roll-out of data collection, lack of time and resources for thorough training and supervision to undertake FGDs and limited coordination with the school leaders before starting the project. The latter may have led to less interest and cooperation from teachers. We were also unable to complete a formal follow-up to measure longer-term learning.

Other studies have also found that youth CS to be acceptable and effective way of collecting scientific data ^32, 33, 37, 62^. One project involving children in a deprived area of Scotland not only engaged children in data collection (on local flora and fauna, waste management, land maintenance, and safety) but in applying their results through dissemination and advocacy. The children created a film which was shown at their school, and to local authorities and political groups, and shared their results with the Scottish government and at the UN Climate Change Conference of the Parties (COP26). These outputs ultimately contributed to improved urban development in their local areas including safer sports places, and new infrastructure linking previously separated areas ^37^. We envisage similar projects could be conducted with school children in Nepal.

In our study, the most significant challenge to implementation of CS in remote mountain areas was lack of internet access. This was particularly problematic with apps that linked into a global community of users ( *e.g.,* iNaturalist). Adolescents also lacked technical skills to create geoshapes on maps of areas affected by hazards. Lack of access to technology, low digital literacy and poor internet connection are common challenges in CS projects in LMICs ^17, 24^. A youth CS study on disaster risk reduction in coastal communities in Brazil overcame difficulties in applying geo-visualisation technology and unreliable internet by adapting their methods to using non-digital alternatives ( *e.g.* printed satellite maps) ^63^. Through these methods they were still able to engage school children to co-create locally contextualised evacuation plans. When apps in our study were limited by lack of internet access, we also found that locally-tailored apps from which data could be manually downloaded and processed by the project team to feed back to the children were more effective.

Research on CS shows that users respond well to simultaneous feedback of data, and tailored health-related messages and recommendations ^20^. Whilst our Nutrition app had this feature, our other bespoke apps collected data that required manual analysis and feedback by a researcher. The pre-existing apps (iNaturalist and OSMTracker) allowed the data collected to contribute to a pooled database with accessible summaries of the data. If automated data processing were built into apps, children and their communities could continue to engage with the data when researchers are not on hand to analyse and feedback data. Future iterations of app development should therefore incorporate both individualised, simultaneous feedback to users, as well as appropriate summaries of data aggregated by user, project or geographical area ( *e.g.,* in the form of a dashboard or summary map).

Data quality and lack of standardised collection methods are a common concern with CS methods ^18, 64^. While adolescents were able to collect a range of climate and health-related data, these were sometimes error-prone and difficult to verify. Supervision by the research team improved data accuracy and reliability whilst collecting nutritional data but the scope for teachers or parents to support the data collection is limited due to lack of general literacy, digital literacy or technical training on the apps and subject matter. Nutritional data collection was feasible, and data could be utilised to gain an understanding of the nutritional status and diets of children five to 19 years old and adults. However, we detected some implausible readings in anthropometric and dietary data. Small sample sizes (especially for children under five years old) limited the utility of the data after implausible data was removed. Dietary diversity results were broadly consistent with other estimates from rural areas of Nepal ^65– 68^. However, when comparing minimum dietary diversity to the 2016 Nepal National Micronutrient Status Survey (NNMSS), our nutritional data has much higher levels of MDD achieved across all age categories compared to the national averages. For example, achievement of MDD in adolescents ranged from 45% (in Jumla) and 66% (in Kavre) in our study compared to national rates of 42-48% in 2016 according to the NNMSS ^66^. The high prevalence of adequate MDD in Kavre could be partially accounted for by inaccuracies in the data collected.

Our data also suggest lower levels of thinness (low BMI Z-for-age) in children five–19 years and lower levels of underweight adults in our samples compared to the national surveys ^66, 67^, which is surprising considering that Jumla is one of the remotest and most food insecure districts in Nepal. A study conducted by Busert *et al.* between 2011 and 2014 in the same municipality of Jumla found that mean dietary diversity of children age zero to 89 months ranged from 3.4 to 3.9, which is lower than the mean of 4.7 found by the citizen scientist adolescents in our study ^65^. Busert *et al.* ^65^ also found extremely high levels of stunting (76.5%) amongst children 2.4 to 7.4 years old, which is three times higher than the levels of stunting (25.5%) found amongst our sample of children aged 14 years on average. While these discrepancies may be partially accounted for by catch-up growth in older children and improving nutrition security since 2013, and our small sample size being unrepresentative, they may also reflect data inaccuracies. To address errors and implausible data in future we recommend larger sample sizes across a representative sampling frame, higher levels of supervision and more training prior to data collection when gathering anthropometric and nutritional data.

Climate hazards were difficult to collect data on as these yielded multiple records of the same data point ( *e.g.* geohazard or failed crops). While a large number of plant species were recorded, data on hazards were too few to measure the full impact of climate change in these regions. Furthermore, the intended purpose of iNaturalist was not always understood by the adolescents, which led to them record domestic animals and well-known crops rather than local wild species. Use of the apps raised adolescents’ awareness of the extent of the biodiversity around them but the links with climate change disrupting habitats and eroding biodiversity were not evident from the data themselves. More extensive data collection over time and monitoring of vulnerable or ‘flagship’ species would be needed to get a measure of changes in biodiversity associated with climate change and to draw conclusions about the impacts of climate change on the local environmental and human health.

While the data may sometimes be less useful for environmental surveillance due to lack of accuracy and reliability, the process was educational and empowering for adolescents, especially when findings were interpreted together with researchers. This finding supports the argument for utilising CS to mobilise collective action on climate change. Increasingly, research in this field is calling for CS to be recognised as a tool not only for scientific research but to promote “legitimate opportunities for citizens to engage with the climate change discourse, define local priorities, and meaningfully influence decisions ^16^.” Few youth CS initiatives include formal advocacy training or capacity building in their project design but those that do demonstrate the value of this in promoting agency for youth climate action ^37^. Due to resource and time constraints this study was unable to provide youth leadership training. Future work should prioritise incorporating youth leadership training to support youth advocacy for uptake of climate change adaptation in their area ^16^. If policy impact is a goal, youth-led CS initiatives should be linked to local policymakers at early stages of project development, since data-based tools should respond to policymaker needs to have the greatest chance of uptake ^69^. It is important during this process, however, not to burden young people with a feeling of responsibility to ‘fix’ climate change issues themselves. For example, it was notable how one adolescent from Jumla discussed how his community could reduce use of motor vehicles when the area is fed by one rough road served by only one or two buses per day. Whilst reducing emissions from polluting old vehicles may be a worthwhile local objective, prioritisation of lobbying for action at regional and national governmental levels would be needed to reduce large scale emissions.

We found that developing a positive relationship with the school principal and teachers was crucial to effective buy-in to our citizen science project. Pilot studies across multiple locations have been vital for scaling up other large CS projects, as participatory approaches require trust and familiarity with local communities before scaling up ^37^. Research has demonstrated the value of relationships built *via* pilot CS projects to provide a foundation for future advocacy and climate resilience work ^63^.

Future research could include scaling up our CS apps both locally and nationally. Applying our method in different climate-vulnerable regions and comparing such approaches between different countries is needed to explore the application in different settings. We envision that the next steps could be to use CS data to involve youth in advocating for locally tailored climate action with local municipal governments and community-based organisations. This might include a large-scale test of the CS approach linked to community groups going through a ‘participatory learning and action’ cycle of meetings to address strategies for climate resilience and conservation agriculture. The impacts on human and environmental health could be measured using a cluster-randomised controlled trial or a step-wedge roll out. After gaining traction at local levels, youth groups could join together regionally and nationally to leverage policy around climate-change and health. Such a programme could build on the strengths of the new decentralised system of government in Nepal whereby municipalities have control over local decision making and spending on development activities. Furthermore, if CS data collection could be scaled up amongst children and communities in Nepal, data could contribute directly to addressing local health impacts of climate change, and even contribute to national or international data sets that could be used to mobilise national and international policy makers. In order to enable a sustainable CS programme in these settings, a local team ( *e.g.* school-led or non-governmental organisation-led) would be needed to maintain continuity and input regularly to support youth in collecting and analysing data in an iterative way and to work with local policy makers. Our lessons learned and recommendations are summarised in Box 1.

Box 1. **Lessons learned and recommendations for future studies.**

- Youth citizen scientists require high levels of supervision from trained facilitators throughout data collection to ensure accurate and reliable data.
- More extensive data collection over a longer time period (e.g. over multiple years including formal follow-up) with monitoring of climate hazards and vulnerable species is needed to measure changes in biodiversity and the impacts of climate change on local environments.
- Large sample sizes collected using a sampling frame should be used where feasible to improve data accuracy, reliability and generalisability.
- Future studies should include comprehensive training on data collection methods with youth CS participants and facilitators and conduct field testing prior to roll-out of data collection.
- Collection of data in the context of an established partnership with local schools increases acceptability and feasibility of the project.
- Access to internet is vital for apps connected to a pooled database; non-digital or manual download alternatives should be considered if applicable.
- Future iterations of app development should incorporate individualised, simultaneous feedback to users and summaries of data aggregated by user, project, or geographical area (e.g., in the form of a dashboard or summary map).
- Collecting data on the process and impact of the CS project on participants (e.g. their perspectives, knowledge and skills, empowerment, and capacity building) in addition to environmental/health data is integral for monitoring the impact and potential for collective climate change action.
- Future youth-led CS initiatives should be linked to local policymakers at early stages of project development and integrate youth leadership training, without burdening adolescents to feel responsible for factors outside their control.

## Conclusion

Our pilot study found that CS methods were an acceptable way to carry out participatory research with adolescents on climate change and nutrition in rural Nepal, although feasibility was limited depending on internet connection. We found that by engaging adolescents in data collection and data interpretation, their knowledge of climate change and nutrition increased. Youth CS methods can be applied to stimulate interest and generate environmental and nutritional data, which could be of value to municipal leaders and health workers and could have applications in future youth advocacy for climate change action. More research is needed on a larger scale to draw conclusions about the quality and utility of the data collected in environmental and health surveillance. Lessons learned from this study can contribute to future work to engage children in influencing policy related to climate change adaptation.

## Data Availability

Figshare: CAP-2030 Nepal: Dataset on sociodemographic characteristics, phone and internet access and climate change awareness: https://doi.org/10.5522/04/22109651 ^54^ This project contains the following underlying data: CAP_Demographics_Jumla_Kavre_recoded.dta CAP_Demographics_Jumla_Kavre_recoded.xls (Data on socio-demographic information about the students and their households including caste/ethnicity, religion, education, water sources, assets, household characteristics, income sources, access to mobile phones or other devices and internet and their concerns with respect to climate change.) Figshare: CAP-2030 Nepal: Dataset on climate-change related hazards: https://doi.org/10.5522/04/22109603 ^55^ This project contains the following underlying data: CAP_Hazard_Kavre_Jumla_varnames.dta CAP_Hazard_Kavre_Jumla_varnames.xls (Data include information, geolocation(latitude/longitude) and/or photos about climate-change associated hazards including landslides, floods, extreme weather events and crop pests/failure, name, and category of the type of hazard, date the hazard event was recorded, date it occurred and the district.) Figshare: CAP-2030 Nepal: Open Street Map tracker mapping dataset: https://doi.org/10.5522/04/22109690 ^56^ CAP2030_OpenStreetMapTracker_dataset_Nepal.dta CAP2030_OpenStreetMapTracker_dataset_Nepal.xls (Data include recorded tracks and way points [latitude/longitude], of categories of waste management [rubbish dumps/bins], water sources, public amenities, and the district.) Figshare: CAP-2030 Nepal: Nutritional dataset (anthropometry and dietary recall): https://doi.org/10.5522/04/22691926 ^61^ This project contains the following underlying data: cap_dietary_combined.dta cap_dietary_combined.xlsx (Nutrition-related data amongst children under 5 years, older children, adolescents and adults: 24-hour dietary recall of healthy food groups and unhealthy sentinel foods, height, weight, BMI, z-scores of height-for-age, weight-for-age, weight-for -height, BMI-for age.) Figshare: CAP-2030 Nepal: Biodiversity datasets: https://doi.org/10.5522/04/22109699 ^57^ This project contains the following underlying data: Data file CAP2030_iNaturalist_dataset1_KavreNepal.xls. Data file CAP2030_iNaturalist_dataset2_KavreNepal.xls. (Data were captured on android tablets using iNaturalist including local names, broad taxa and probable species, geolocation and time/ date of capture.) Figshare: CAP-2030 Nepal: Focus Group Discussions on youth citizen science about climate change and nutrition: https://doi.org/10.5522/04/22109714 ^70^ This project contains the following underlying data: Climate_change_FGDs_CAP2030_Nepal_clean.docx. (Transcripts of six Focus Group Discussions [FGDs] conducted with adolescent school students in Nepal about citizen science and climate change: Four FGDs conducted in June 2022 in Jumla district in remote West Nepal and two FGDs conducted in May 2022 with in Kavre district in Central Nepal 2 hours’ drive from Kathmandu. Data were audio recorded, transcribed into Nepali, and translated into English. The first two focus groups cover Jumla students’ pre-existing knowledge of and concerns about climate change, and their access to and experience of using mobile devices. The last four focus groups cover Jumla and Kavre students’ responses to the “CAP2030 Nepal” citizen science project where they had the opportunity to learn to collect data on mobile devices [tablets] about climate hazards [ *e.g*., landslides, floods and failed crops], waste and water management, biodiversity in their locality, and diets and nutritional status of the local community.) Figshare: CAP-2030 Nepal: Dataset on plant diversity in Jumla district (raw data): https://doi.org/10.5522/04/22109618 ^60^ This project contains the following underlying data: CAP2030_Plant_Atlas_Jumla_2022-06-20-19-18-13_labelled.dta CAP2030_Plant_Atlas_Jumla_2022-06-20-19-18-13_labelled.xls (Data includes the latitude/longitude, name of the plant species and category [crop, wild], date it was recorded, and the district.) Figshare: CAP2030 project Biodiversity photographs collected using iNaturalist app in Nepal. https://doi.org/10.5522/04/22109417 ^58^ This project contains the following extended data: Photos of various wild and cultivated plants in Jumla district of Nepal captured on android tablets using iNaturalist. Figshare: CAP2030 project Biodiversity photographs collected using iNaturalist app in Nepal. https://doi.org/10.5522/04/22109555 ^59^ This project contains the following extended data: Photos of various insects and other invertebrates, reptiles, amphibians, birds and mammals (including some domesticated birds and mammals) and algae in Jumla district of Nepal captured on android tablets using iNaturalist. Data are available under the terms of the Creative Commons Zero “No rights reserved” data waiver (CC0 1.0 Public domain dedication). Source code available from: https://github.com/Bhawak/CS-collect/tree/master/CAP2030-forms https://github.com/Bhawak/CS-collect Archived source code at time of publication: https://doi.org/10.5281/zenodo.7762177 ^47^ License: Apache License Version 2.0 Source code available from: https://github.com/Bhawak/OSMTrackerAndroid Archived source code at time of publication: https://doi.org/10.5281/zenodo.7762181 ^45^ License: GNU Lesser General Public License Version 3 Source code available from: https://github.com/Bhawak/iNaturalistCAP2030Nepal Archived source code at time of publication: https://doi.org/10.5281/zenodo.7762183 ^46^ License: MIT
