# Supplementary material for: Children in All Policies (CAP) 2030 Citizen Science for Climate Change Resilience: a cross-sectional pilot study engaging adolescents to study climate hazards, biodiversity and nutrition in rural Nepal
Source: Wellcome Open Res. 2023 Dec 11;8:570. [Version 1] doi: 10.12688/wellcomeopenres.18591.1 (PMC10904941; doi:10.12688/wellcomeopenres.18591.1)
Supplement: Peer review history

# Reviewer report: Reviewer response for version 1

**DOI:** 10.21956/wellcomeopenres.20616.r71259

**Published:** 2024-02-26

**Authors:** Sylvia I. Bergh

**Affiliations:** 1 The Hague University of Applied Sciences, The Hague, Netherlands Antilles

This is a very interesting and well-written article on the experiences of having young people in rural Nepal collect data to track climate hazards, biodiversity and nutrition. While it is quite limited in its set-up, the experience and lessons learnt are well documented and therefore useful for other researchers interested in doing similar action-research projects in developing countries.

 I have some minor suggestions on how to improve the article further.

- First, the way the third research question is formulated (asking about the extent to which Nepali adolescents were aware about climate change issues before and after participating in the citizen science activities, p. 4) would imply a survey-set up to be able to answer this question with some accuracy. However, Table 3 only reports prior climate change awareness (and the choice seems to only have been between 'knows something' and 'knows very little') but I could not find such a report for the end of the project. While there are some relevant qualitative findings on this point as well, perhaps the question could be reformulated to make it less quantitative.
- Second, it could be explained more why it was necessary to use seven different apps - why not integrate their functionalities into one single app? This would have made it easier for the less experienced users and increased the time available for data collection rather than time spent on learning how to use the apps.
- Third, for ethical reasons, it would have been useful to clarify whether the donating of the 10 tablets to the school in Jumla at the end of the project was announced to the local trainers from the start or not. This would help the reader to know whether this expectation might have influenced their decision to participate.
- Fourth, the Micro-Poll project mentioned on p. 12 seems to have served as a kind of control group, and thus could have been introduced in more detail. These are quite minor suggestions on an otherwise very worthwhile study.

Is the work clearly and accurately presented and does it cite the current literature?

Yes

If applicable, is the statistical analysis and its interpretation appropriate?

Not applicable

Are all the source data underlying the results available to ensure full reproducibility?

Yes

Is the study design appropriate and is the work technically sound?

Yes

Are the conclusions drawn adequately supported by the results?

Yes

Are sufficient details of methods and analysis provided to allow replication by others?

Yes

Reviewer Expertise:

Citizen science, sustainability, climate change, development studies

I confirm that I have read this submission and believe that I have an appropriate level of expertise to confirm that it is of an acceptable scientific standard.

# Reviewer report: Reviewer response for version 1

**DOI:** 10.21956/wellcomeopenres.20616.r71261

**Published:** 2024-02-14

**Authors:** Ana Bonell

**Affiliations:** 1 MRC Unit The Gambia, London School of Hygiene and Tropical Medicine, Fajara, The Gambia

Thank you for asking me to review this paper which describes a pilot study of citizen science engagement of adolescents living in Nepal on the interaction between climate, biodiversity and human health. It is a pleasure to read this very well-written and thorough piece of work. 

 The objectives of the paper are clearly set out, the methods explained well and the results are given in great detail. The conclusions are in keeping with the findings from the study.

 The paper is complete as is, although I would suggest one minor addition. For the final objective (awareness on climate change issues before and after participating) it would be interesting to know if this was evaluated quantitatively. It is well described in the qualitative findings, but would be good to complement this with quantitative findings.

Is the work clearly and accurately presented and does it cite the current literature?

Yes

If applicable, is the statistical analysis and its interpretation appropriate?

Yes

Are all the source data underlying the results available to ensure full reproducibility?

Yes

Is the study design appropriate and is the work technically sound?

Yes

Are the conclusions drawn adequately supported by the results?

Yes

Are sufficient details of methods and analysis provided to allow replication by others?

Yes

Reviewer Expertise:

climate change and human health

I confirm that I have read this submission and believe that I have an appropriate level of expertise to confirm that it is of an acceptable scientific standard.
